# Fast and Sensitive Detection of Anti-SARS-CoV-2 IgG Using SiO_2_@Au@CDs Nanoparticle-Based Lateral Flow Immunoassay Strip Coupled with Miniaturized Fluorimeter

**DOI:** 10.3390/biom14121568

**Published:** 2024-12-09

**Authors:** Rui Wang, Junping Xue, Guo Wei, Yimeng Zhang, Chuanliang Wang, Jinhua Li, Xuhui Geng, Abbas Ostovan, Lingxin Chen, Zhihua Song

**Affiliations:** 1 School of Pharmacy, Collaborative Innovation Center of Advanced Drug Delivery System and Biotech Drugs in Universities of Shandong, Key Laboratory of Molecular Pharmacology and Drug Evaluation (Yantai University), Ministry of Education, Yantai University, Yantai 264005, China; wr17606453912@yeah.net (R.W.); xjp199808@yeah.net (J.X.); guovv713@yeah.net (G.W.); zym19990110@yeah.net (Y.Z.); 2 Department of Instrumentation & Analytical Chemistry, CAS Key Laboratory of Separation Sciences for Analytical Chemistry, Key Laboratory of Deep-Sea Composition Detection Technology of Liaoning Province, Dalian Institute of Chemical Physics, CAS, 457 Zhongshan Road, Dalian 116023, China; wangcl@dicp.ac.cn (C.W.); xhgeng@dicp.ac.cn (X.G.); 3 CAS Key Laboratory of Coastal Environmental Processes and Ecological Remediation, Research Center for Coastal Environmental Engineering and Technology, Yantai Institute of Coastal Zone Research, Chinese Academy of Sciences, Yantai 264003, China; jhli@yic.ac.cn (J.L.); a.ostovan@yic.ac.cn (A.O.); lxchen@yic.ac.cn (L.C.)

**Keywords:** SiO_2_@Au@CDs nanoparticles, lateral flow immunoassay, SARS-CoV-2 IgG antibody, fluorescence measurement, high sensitivity and rapidity

## Abstract

The development of a novel strategy for the measurement of SARS-CoV-2 IgG antibodies is of vital significance for *COVID-19* diagnosis and effect of vaccination evaluation. In this investigation, an SiO_2_@Au@CDs nanoparticle (NP)-based lateral flow immunoassay (LFIA) strip was fabricated and coupled with a miniaturized fluorimeter. The morphology features and particle sizes of the SiO_2_@Au@CDs NPs were characterized carefully, and the results indicated that the materials possess monodisperse, uniform, and spherical structures. Finally, this system was employed for SARS-CoV-2 IgG antibody test. In this work, the strategy for the SARS-CoV-2 IgG antibody test possesses several merits, such as speed (less than 15 min), high sensitivity (1.2 × 10^−7^ mg/mL), broad linearity range (7.4 × 10^−7^~7.4 × 10^−4^ mg/mL), accurate results, high selectivity, good stability, and low cost. Additionally, future trends in LFAs using quantum dot-based diagnostics are envisioned.

## 1. Introduction

Coronavirus disease 2019 (*COVID-19*), a newly severe acute respiratory disease, is caused by SARS-CoV-2 (a severe acute respiratory syndrome coronavirus 2) and was first reported in early December 2019 [1,2]. By the end of August 2023, *COVID-19* had affected human health and seriously threatened life, with more than 7.6 hundred million infections and 6.9 million deaths, while the world economy declined sharply. In view of this, rapid and accurate diagnosis is a very effective strategy to limit the spread of SARS-CoV-2 and suppress *COVID-19* [3]. Thus, the use and evaluation of the properties of vaccines are particularly necessary. Currently, there are two essential diagnostic approaches for *COVID-19*: nucleic acid measurement (reverse transcription-quantitative polymerase chain reaction, RT-qPCR) and serological assay (such as enzyme immunoassay (EIA), lateral flow immunoassays (LFIAs), chemiluminescent immunoassay (CLIA), and microsphere-based antibody assays) [4]. Among them, traditional RT-qPCR, owing to its advantages of early discovery and ultra-sensitive testing, has been the most widely used method and is regarded as the “gold standard method” for diagnosing *COVID-19* [5,6]. However, RT-qPCR diagnostics for *COVID-19* possess limitations such as the need for expensive instruments, inadequate access to reagents, high false-negative rates, the need for professional technicians, and time-consuming procedures (>24 h) [4,6,7]. Nevertheless, some areas cannot meet the standard of RT-qPCR diagnostics [8]. Therefore, it is quite necessary to develop supplementary approaches to diagnose *COVID-19*.

Nowadays, immunoglobulin-G (IgG) and immunoglobulin-M (IgM) antibodies are regarded as reliable tools for diagnosing whether a person has been affected by SARS-CoV-2 [9]. Commonly, the contents of IgM and IgG increase sharply 4 days and 12 days after symptom onset, respectively [10]. Thus, the state of the infection can be revealed by the IgG content [11]. Additionally, the contents of IgG and IgM in vivo are important parameters to evaluate the effect of the *COVID-19* vaccine, and the measurement of IgG and IgM is critical to the determination of vaccination interval. At present, many approaches have been developed to measure IgG and IgM antibodies. Enzyme-linked immunosorbent assay (ELISA), possessing the advantages of flexibility, high throughput, and multiple sample testing, is a primary approach for the measurement of various antibodies (including IgG, IgM, IgA, and many other kinds of antibodies) [12]. Zheng et al. utilized the ELISA method to evaluate IgG and IgM antibodies and verified the excellent sensitivity of ELISA [13]. More recently, Song et al. detected SARS-CoV-2 IgM/IgG in 200 people without *COVID-19* infection (antinuclear antibody (ANA), rheumatoid factor (RF) positive group, pregnant women group, positive group, and normal senior group) using the ELISA strategy. The content of SARS-CoV-2 IgG in the normal control group was lower than that in the pregnant women, and no obvious difference was found between other groups [14]. ELISA possesses the advantages of high sensitivity, rapid detection, and low cost, and can detect febrile patients effectively [15], and was promised to be an alternative approach to RT-qPCR for *COVID-19* diagnosis. Although ELISA is very essential in controlling the disease, it is time-consuming (taking about 245 min) and suffers from the deficiency of low sensitivity [16,17]. A rapid and accurate method for diagnosing is urgently warranted [18].

LFIAs, belonging to immunochromatographic assays or lane tests, have gained tremendous attention for the detection of IgG and IgM antibodies owing to their remarkable properties (including simplicity, cost-effectiveness, good stability, effortless production, and rapid time-to-result) [12,19]. Therefore, LFIAs are considered a point-of-care (POC) diagnostic strategy. As early as 1917, LFIAs were being used to diagnose infectious disease by Dochez and Avery and could be used to analyze pneumococcal polysaccharides from the urine and serum of patients with lobar pneumonia [20]. Nowadays, LFIAs perform well in the analysis of IgM and IgG antibodies, antigens, and RNA [21]. In 2020, Li’s group first applied LFIAs for the determination of SARS-CoV-2. They finished the detection process in 15 min, and the resulting testing sensitivity and specificity were 88.7% and 90.6%, respectively [22]. After that, Adams et al. utilized 12 LFIAs to measure SARS-CoV-2 antibodies, sensitivity against RT-PCR from 30.3% to 74% for IgG, 21.2~67% for IgM, and 37.4~79% for IgM/IgG were obtained; furthermore, specificity and sensitivity were from 85.4 to 100% and 44.6% to 95.4%, separately [23]. Recently, LFIAs have been used to test SARS-CoV-2 IgG with a high confidence interval (95%), and the disease risk in unvaccinated individuals could be predicted before 200 days [24]. However, LFIAs have inadequacies, including insensitivity and difficulties with the accurate quantification of antibodies, antigens, and RNA [25].

Over the years, numerous fluorescence-based LFIAs have been proposed for anti-SARS-CoV-2 IgG detection. Up to now, varieties of nanomaterials have been utilized to enhance the sensitivity of *COVID-19* diagnosis [26]. Silica nanoparticles (NPs), possessing the properties of good stability and compatibility with various matrices, have been employed as carrier materials in several fields (sample pretreatment, bioanalysis, catalysis, etc.) [27,28]. Quantum dots (QDs) have drawn scientific attention owing to their optoelectronic properties [29]. Carbon dots (CDs), a novel kind of carbon NP (particle size less than 10 nm), have been used as fluorescent materials by many researchers because of their advantages (such as perfect fluorescence properties, good conductivity, superior biocompatibility, excellent water solubility, high chemical stability, easy synthesis procedure, cost-effectiveness, good environmental protection, and nontoxicity) [30,31]. Au nanoparticles (Au NPs) have been extensively used due to their highly attractive features including localized surface plasmon resonance (light can be captured by Au NPs with high efficiency and converted into heat), nano-size, low toxicity, easy functionalization, and chemical inertness [32,33,34].

It is of vital importance to develop a rapid, reliable, and cost-effective strategy to detect SARS-CoV-2 IgG. Lanthanide-doped polysterene nanoparticle (LNP)-based LFIAs have never been applied for anti-SARV-CoV-2 IgG detection, which could satisfy the requirements of clinical diagnostic reagents [35]. Additionally, other compounds (other than QDs) have never been used for anti-SARS-CoV-2 IgG antibody determination. Long et al. employed AF680-labeled secondary antibody as a fluorescence signal reporter for quantification of anti-SARS-CoV-2 IgG antibody in serum, and the test time was about 25 min. The linearity range and low limit of detection (LOD) were 12.5–1000 ng/mL and 12.5 ng/mL, respectively [36]. After that, the research group of Aznar utilized Rhodamine B (RhB) as a fluorescence probe for SARS-CoV-2 N protein IgG antibody determination. This system possessed high selectivity and an LOD of 1 µg/mL, and the detection procedure could be finished in 20 min [37]. Nevertheless, it is urgently needed to improve selectivity, linearity range, and test time.

Herein, SARS-CoV-2-specific IgG was detected using QDs-doped lateral flow immunoassays coupled with a miniaturized fluorimeter, and SiO_2_@Au@CDs NPs were utilized as fluorescent materials (the QDs were synthesized using silica NPs as the core, Au NPs were coated on the surface of silica NPs, and CDs were modified on the outermost layer), which could combine the localized surface plasmon resonance (LSPR) of Au NPs and the good fluorescence property of CDs. What’s more, a miniaturized fluorimeter was applied, and the mechanism of fluorescence enhancement resulting from metal particles and CDs was utilized. The system (SiO_2_@Au@CDs nanoparticle-based LFIA strips coupled with a miniaturized fluorimeter) possessed high sensitivity (QDs and a miniaturized fluorimeter) and high selectivity (SARS-CoV-2 S1 protein-labeled SiO_2_@Au@CDs NPs towards SARS-CoV-2 IgG). This system is very suitable for SARS-CoV-2-specific IgG detection and reveals potential clinical applications.

## 2. Experimental Section

### 2.1. Reagents

Bovine serum albumin (BSA), tritonX-100, and rabbit anti-mouse IgG were provided by Beijing Solarbio Science&Technology Co., Ltd. (Beijing, China). Saccharose (analytically pure) was purchased from Xilong Scientific Co., Ltd. (Shantou, China). Carbon dots (CDs) were obtained from Jiangsu XFNANO Materials Tech. Co., Ltd. (Nanjing, China). SARS-CoV-2 spike S1 protein and anti-SARS-CoV-2 spike antibody were purchased from Wuhan Huamei Biotech. Co., Ltd. (Wuhan, China). MERS-CoV IgG1 (Catalog#40068-MM10), RSV IgG (Catalog#11070-MM15), and ASF1B IgG (Catalog#14382-MM02) were obtained from Sino Biological Inc. (Beijing, China). Polyethyleneimine (PEI), 2-(N-Morpholino)ethanesulfonic acid monohydrate (MES), monopotassium phosphate, trisodium citrate (TSC) and 1-(3-Dimethylaminopropyl)-3-ethylcarbodiimide hydrochloride (EDC) were purchased from Shanghai Aladdin Biochemical Technology Co., Ltd. (Shanghai, China). Disodium hydrogenorthophosphate, tween 20, N-Hydroxysulfosuccinimide sodium, and potassium carbonate were bought from Energy Chemical Co., Ltd. (Shanghai, China). Poly(vinyl alcohol) (PVA, MW 40000), HAuCl_4,_ and polyvinylpyrrolidone (PVP) were from Shanghai Yuanye Bio-Technology Co., Ltd. (Shanghai, China). Sodium borohydride was purchased from Sigma-Aldrich. Methyl alcohol (MeOH), ethyl alcohol (EtOH), and sodium hydroxide (NaOH) were obtained from Sinopharm Chemical Reagent Beijing Co., Ltd. (Beijing, China). Silica NPs were from Suzhou Guangduo Electronic Materials Co., Ltd. (Suzhou, China). Glass fiber conjugate pads, cellulosic sample pads, and Nitrocellulose (NC) membranes were from Shanghai Jieyi Biotechnology Co., Ltd. (Shanghai, China). Rats were purchased from Jinan Pengyue Experimental Animal Breeding Co., Ltd. (Jinan, China). The ultrapure water from Wahaha Group Co., Ltd. (Hangzhou, China) was utilized in the experiment.

### 2.2. Instruments

Transmission electron microscope (TEM, JEM-1400plus, JEOL, Tokyo, Japan) and scanning electron microscopy (SEM, S-4800, HITACHI, Tokyo, Japan) were utilized to measure and analyze the morphology of the materials. The energy dispersive spectroscopy (EDS) spectra of the materials were acquired through SEM equipped with an EDS. Additionally, the mass percent of carbon (C) and nitrogen (N) in the materials were measured by an elemental analyzer (vario MACRO cube, Elementar Analysensysteme GmbH, Langenselbold, Germany). Zetasizer Nano ZS (Marlvern MS2000, Malvern Panalytical, UK) was used to detect the particle size distribution and zeta potential of the materials. Fluorescence spectra and ultraviolet–visible (UV-Vis) absorption spectra measurement of the materials was carried out by using a fluorescence spectrophotometer (F-4700, HITACHI, Tokyo, Japan) and ultraviolet spectrophotometer (UV-1900, Shimadzu, Kyoto, Japan), respectively. A miniaturized fluorimeter (excitation wavelength: 365 nm, emission wavelength: 605 nm) was obtained from the Dalian Institute of Chemical Physics, Chinese Academy of Sciences (Dalian, China).

### 2.3. Preparation of Au NPs

The Au NPs were synthesized according to the strategy proposed by Wang et al. [3], in short, a sodium borohydride reduction method was employed. 100 mL of aqueous solution containing TSC solution (1 mL, 1% (*w*/*w*)) and HAuCl_4_ solution (1 mL, 1% (*w*/*w*)) was stirred intensely. After that, the aqueous solution containing NaBH_4_ (1.5 mL, 0.2 mol/L) was mixed with the above solution, and the mixture kept stirring for 4 h. Finally, the resulting ~4 nm Au NPs were refrigerated at 4 °C for later use.

Meanwhile, Au NPs with a particle size of about 40 nm were prepared via the TSC reduction approach. Firstly, HAuCl_4_ solution (0.05 mL, 1% (*w*/*w*)) was added into ultrapure water (4.95 mL), the solution was heated and kept boiling for 2 min. Secondly, TSC solution (0.055 mL, 1% (*w*/*w*)) was added to the above mixture solution, and the mixture was heated to reflux for 15 min under magnetic stirring. In the end, the mixture containing ~40 nm Au NPs was stored at the same condition as that of ~4 nm Au NPs mentioned above.

### 2.4. Synthesis of Sandwich-Like SiO_2_@Au@CDs NPs

In this work, PEI was utilized as a linker, and the positively charged materials resulted after coating PEI on the surface of silica NPs via electrostatic adsorption interaction. The method for preparation of sandwich-like SiO_2_@Au@CDs NPs is as follows: 0.25 mL of 1 mmol/L SiO_2_ NPs and 20 mL of 0.5 mg/mL PEI solution were mixed, the mixture was sonicated for half an hour; then, 5 mL of the SiO_2_@PEI NPs and 50 mL of 4 nm Au NPs were mixed together under ultrasonic condition. Half an hour later, SiO_2_@Au NPs were obtained via centrifuge for 6 min and dispersed in ultrapure water (2.5 mL). After that, the resulting SiO_2_@Au NPs were added into 25 mL of 0.5 mg/mL PEI solution, SiO_2_@Au@PEI material was obtained after sonication for an hour. Next, SiO_2_@Au@PEI NPs and CDs (0.2 mL, 1 mmol/L) were mixed and sonicated for half an hour. In the end, SiO_2_@Au@CDs NPs were obtained after 6000 rpm centrifugation for 6 min, and resuspended in ethanol (5 mL) for later use.

### 2.5. Synthesis of SARS-CoV-2 S1 Protein-Labelled SiO_2_@Au@CDs NPs

In this study, SARS-CoV-2 S1 protein-labeled SiO_2_@Au@CDs NPs were synthesized via carbodiimide chemistry strategy. Briefly, SiO_2_@Au@CDs NPs (1 mL) were collected by centrifugation and dispersed in the buffer solution containing 2-morpholinoethanesulfonic acid (MES) (1 mL, 0.1 mol/L, pH 5.5); the solution of EDC (10 μL, 0.1 mol/L) and sulfo-NHS (20 μL, 0.1 mol/L) were added; the reaction was carried out under ultrasonic conditions for 15 min. Unreacted EDC/sulfo-NHS was removed by centrifugation under 6000 rpm for 6 min, and the material was dispersed in phosphate-buffered saline (PBS) (200 μL, 0.01 mol/L, pH 7.4). Subsequently, SARS-CoV-2 S1 protein (10 μg) was added, and the reaction was carried out for 2 h at 25 °C under shaking (200 rpm). BSA (250 μL, 3%) was used to block the nonreacting carboxyl groups on the SiO_2_@Au@CDs NPs surface. In the end, SARS-CoV-2 S1 protein-labeled SiO_2_@Au@CDs NPs were washed twice by PBS buffer (0.01 mol/L, pH 7.4) containing the mixture solution (0.05% Tween 20 (Polysorbate 20) (*v*/*v*), 1% BSA (*w*/*v*), 0.1% PVP (*w*/*v*), and 10% sucrose (*w*/*v*, g/mL)) and collected by centrifugation under 6000 rpm for 6 min. The resulting SARS-CoV-2 S1 protein-labeled SiO_2_@Au@CDs NPs were dispersed in PBS buffer (300 μL, 0.01 mol/L, pH 7.4) for later use. Finally, the conjugate pad coated with SARS-CoV-2 S1 protein-labeled SiO_2_@Au@CDs NPs was obtained by dropping the liquid onto the blank conjugate pad and vacuum freeze-drying. Meanwhile, a control experiment was conducted by replacing the SARS-CoV-2 S1 protein (10 μg) with BSA (10 μg), and the resulting BSA-modified SiO_2_@Au@CDs NPs were used as a comparison.

### 2.6. Preparation of LFIA Strip

The LFIA strip (consisting of four parts: an NC membrane marked with a test line (T line) and a control line (C line), an absorption pad, a conjugate pad, and a sample pad) was prepared for detection of SARS-CoV-2-specific IgG. The T line and C line were prepared by spraying 1.05 mg/mL of rabbit anti-mouse IgG antibody (secondary antibody) and 7.4 mg/mL of anti-SARS-CoV-2 IgG antibody onto the NC membrane, separately. Then, the NC membrane was kept in an oven (37 °C) for three hours. After that, the NC membrane with a T line and a C line was assembled onto a clean plate (PVC). The absorption pad and conjugate pad were assembled onto the plate near the C line and T line (the distance between the C line and T line was 4~6 mm), respectively; and a distance of 2 mm was overlapped by the two pads to keep the dropped sample moving fast. The integrated membranes were pressed for 5~10 min, and they were trimmed to strips with a width of 3 mm. Finally, the LFIAs were obtained by cutting the plates into individual strips (width: 5 mm), and stored in dry, sealed, and cool (4 °C) conditions for later detection.

### 2.7. Detection of SARS-CoV-2-Specific IgG by LFIA Strip

50 μL sample (including 1 μL of rat serums spiked with anti-SARS-CoV-2 IgG antibody) was added into 100 μL of PBS buffer (0.01 mol/L, pH 7.4), and the mixture was dropped onto the sample pad. S1 protein-conjugated labels could be captured by the T line and C line when the sample moved from the sample pad to the absorbent pad. Fifteen minutes later, the turn colors and fluorescence of the T line were observed by the naked eye and measured by a miniaturized fluorimeter, respectively. The concentration of the SARS-CoV-2-specific IgG was calculated by making an equation of linear regression.

## 3. Results and Discussion

### 3.1. Synthesis and Characterization of SiO_2_@Au@CDs NPs

**Figure 1:**
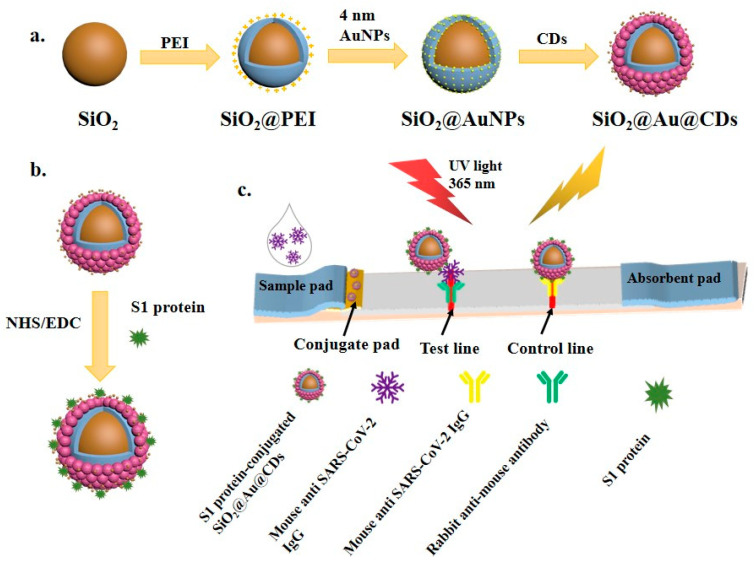
Schematic diagram for (**a**) synthesis of SiO_2_@Au@CDs NPs; (**b**) preparation of S1 protein-labeled SiO_2_@Au@CDs NPs; (**c**) fabrication of LFIA.

As shown in Figure 1a, SiO_2_@Au@CDs NPs were prepared by using commercial silica NPs as the core. PEI was used as the linker based on electrostatic interaction, and Au NPs and CDs were coated on the surface of silica NPs using a layer-by-layer strategy. Au NPs and CDs were devoted to supplying fluorescence signals. After that, S1 protein-labeled SiO_2_@Au@CDs NPs were synthesized by using SARS-CoV-2 S1 protein as the marker protein (as illustrated in Figure 1b). Finally, LFIA strips (illustrated in Figure 1c) were fabricated in four parts: a sample pad, a conjugate pad (providing dual-mode label loading), an NC membrane (consisting of T line and C line), and an absorbent pad (providing capillary force).

**Figure 2:**
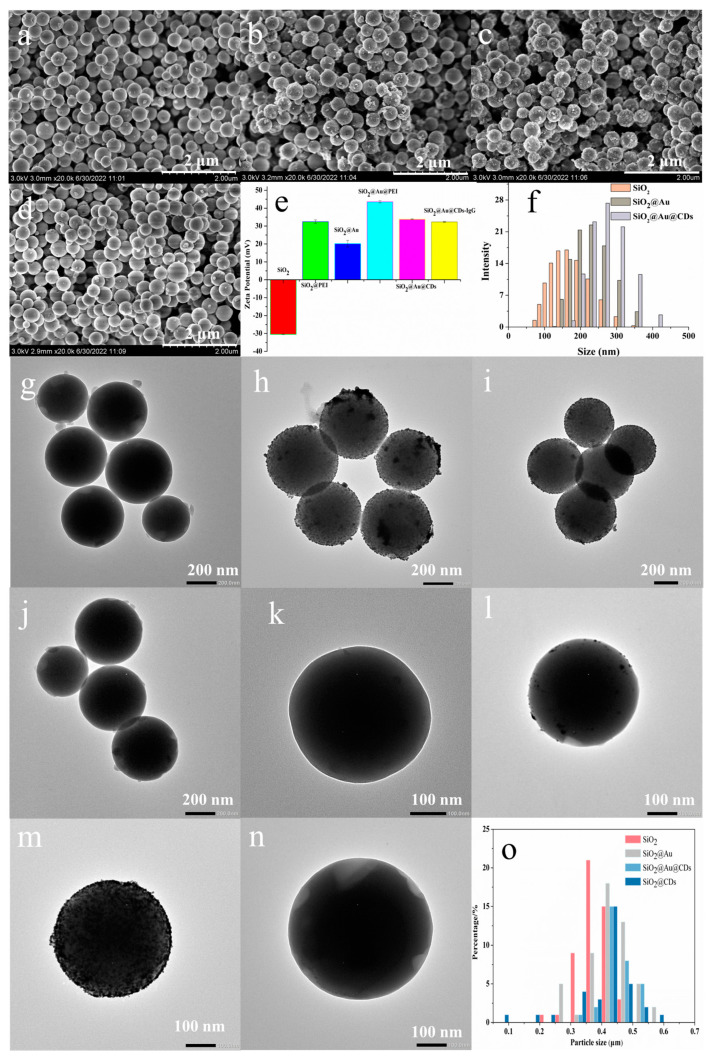
Characterization of the NPs. SEM images: (**a**) SiO_2_; (**b**) SiO_2_@Au; (**c**) SiO_2_@Au@CDs; (**d**) SiO_2_@CDs; (**e**) zeta potential of the materials; (**f**) particle size distribution of the materials; TEM images: (**g**,**k**) SiO_2_, (**h**,**l**) SiO_2_@Au, (**i**,**m**) SiO_2_@Au@CDs, (**j**,**n**) SiO_2_@CDs; (**o**) particle size distribution histograms of the materials via TEM.

In this work, the morphology, microstructure, particle size distribution, zeta potential, elements composition, and optical properties of the resulting sandwich-like SiO_2_@Au@CDs NPs were characterized and analyzed by SEM, TEM, Zeta sizer Nano ZS, EDS, elemental analyzer, F-4700, and UV-1900, respectively. As exhibited in Figure 2a–d, the surfaces of SiO_2_@Au, SiO_2_@CDs, and SiO_2_@Au@CDs NPs were rougher than those of bare silica NPs, which demonstrates the successful coating of Au NPs and CDs layers. Additionally, the SiO_2_@Au@CDs NPs have clear profiles and a monodisperse, uniform, and spherical structure. Meanwhile, as illustrated in Figure 2e, the zeta potential of the NPs increased significantly with the PEI coating and decreased with the coating of Au NPs and CDs, which further confirmed the existence of electrostatic interaction. SARS-CoV-2 S1 protein-labeled SiO_2_@Au@CDs NPs were immersed into IgG solution, and then NPs were washed thrice using ultrapure water. The zeta potential of the IgG-attached SARS-CoV-2 S1 protein-labeled SiO_2_@Au@CDs NPs solution was +32.3 mV, lower than that without IgG attachment (+33.8 mV), which indicated the successful attachment of IgG upon the surface of NPs. Furthermore, as shown in Figure 2f, the particle sizes of bare silica NPs, SiO_2_@Au NPs, and SiO_2_@Au@CDs NPs were mainly near 160 nm, 230 nm, and 270 nm, separately, indicating the successful coating of the nanolayer. The results were further confirmed by TEM. As shown in Figure 2g–n, the NPs were monodispersed, and they showed unobvious aggregation. Several NPs were successfully fabricated on the surfaces of the materials. As shown in Figure S1 and Table S1, Au was detected in SiO_2_@Au and SiO_2_@Au@CDs. What’s more, in Figure 2o, the particle sizes of the materials increased with the coating of Au NPs and CDs. As shown in Table S2, elements including C and N could be detected in SiO_2_@CDs, SiO_2_@Au, and SiO_2_@Au@CDs. The results reveal these materials have been successfully synthesized.

Furthermore, the optical properties (including UV-Vis absorption spectra and fluorescence spectra) of the materials were investigated meticulously. As depicted in Figure S2, the absorption peaks of Au NPs (4 nm), SiO_2_@Au, and SiO_2_@Au@CDs were about 510 nm, 520 nm, and 600 nm, respectively. The reason for this is the plasmon resonance excitation wavelength of the 4 nm Au NPs. In this investigation, Au NPs and CDs were utilized as plasma and fluorophore, separately. The local electromagnetic field surrounding Au NPs could be enhanced, and the fluorescence intensity of nearby CDs could be finally enhanced, which was termed metal-enhanced fluorescence [38,39,40,41]. Enhancement effects are mainly dependent on the distance between metal NPs and QDs, and the particle size of metal NPs [38,39,40,41]. As shown in Figure S3, the excitation wavelength of 365 nm was used to measure the fluorescence spectra of the materials. No fluorescence emission of SiO_2_ and SiO_2_@Au NPs could be observed, which was because Au NPs have no fluorescent properties. The fluorescence intensity of SiO_2_@Au@CDs NPs was higher than that of SiO_2_@Au NPs and SiO_2_@CDs NPs owing to the plasmon nature of Au NPs.

These further confirmed the appropriate optical properties of the SiO_2_@Au@CDs NPs. Their fluorescence characteristics were of particular importance to achieve both high sensitivity and reliability in anti-SARS-CoV-2 IgG detection.

### 3.2. Structure and Principle of LFIA for Anti-SARS-CoV-2 IgG Detection

As depicted in Figure 1c, the principle of LFIA for anti-SARS-CoV-2 IgG detection was as follows: first, the sample was dropped onto the sample pad, and moved through the conjugate pad, T line, and C line based on capillary force from the absorbent pad. In the process, the samples containing anti-SARS-CoV-2 IgG were associated with SARS-CoV-2 S1 protein-labeled SiO_2_@Au@CDs NPs by antibody–antigen interaction. Then, the complex moved, and they were captured quickly by anti-IgG antibodies on the T line. The extra SARS-CoV-2 S1 protein-labeled SiO_2_@Au@CDs NPs were absorbed by the C line (anti-SARS-CoV-2 IgG antibody). After that, the T line was measured by the naked eye and a miniaturized fluorimeter. The C line was used to estimate the usability of the LFIA strip. The intensity of the fluorescence signal (measured by a miniaturized fluorimeter) was proportional to the anti-SARS-CoV-2 IgG concentration, and this is of vital importance for the quantitative analysis of anti-SARS-CoV-2 IgG. The pictures of this fluorescence detection device and the relevant video of the measurement process have been attached in Figure S4 and Video S1, respectively.

**Figure 3:**
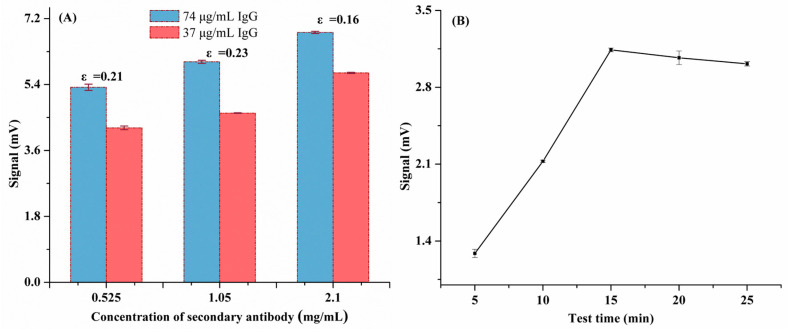
(**A**) Effect of secondary antibody concentration; (**B**) different testing time of LFIA for IgG.

Researchers have confirmed that the SARS-CoV-2 S1 protein possesses superior specificity and immunogenicity for *COVID-19* diagnosis [42,43]. As shown in Figure 1b, SARS-CoV-2 S1 protein-labelled SiO_2_@Au@CDs NPs were synthesized via the EDC/NHS-based covalent coupling between COOH groups of SiO_2_@Au@CDs and SARS-CoV-2 S1 protein. First, as demonstrated in Figure 3A, the concentration of secondary antibodies (0.525, 1.05, and 2.1 mg/mL) on the T line was optimized. The sensitivity index (ε = (S2 − S1)/S2, S1 was the fluorescence signal of 37 ng/mL, and S2 was the fluorescence signal of 74 ng/mL) was utilized to optimize the concentration of the secondary antibody. In the process, the higher the ε, the better the sensitivity [36]. Thus, the optimal secondary antibody concentration of 1.05 mg/mL was used throughout the subsequent investigation.

In addition, for anti-SARS-CoV-2 IgG (7.4 × 10^−4^ mg/mL) detection, the fluorescence intensity values of BSA-modified SiO_2_@Au@CDs NP-based LFIA and SARS-CoV-2 S1 protein-labeled SiO_2_@Au@CDs NP-based LFIA were 1207 μv and 6102 μv, respectively, which revealed that the BSA could not detect the same IgG. The results confirmed that the SARS-CoV-2 S1 protein-labeled SiO_2_@Au@CDs NP-based LFIA possessed outstanding selectivity towards anti-SARS-CoV-2 IgG analysis. Furthermore, the assay time was explored, as exhibited in Figure 3B; with the assay time increasing, the fluorescence signal enhanced obviously; however, after 15 min, a slight decrease was observed. Therefore, 15 min was chosen as the optimal assay time for anti-SARS-CoV-2 IgG detection in the subsequent experiments.

### 3.3. Evaluation of Quantum Dot-Doped LFIA Strip

**Figure 4:**
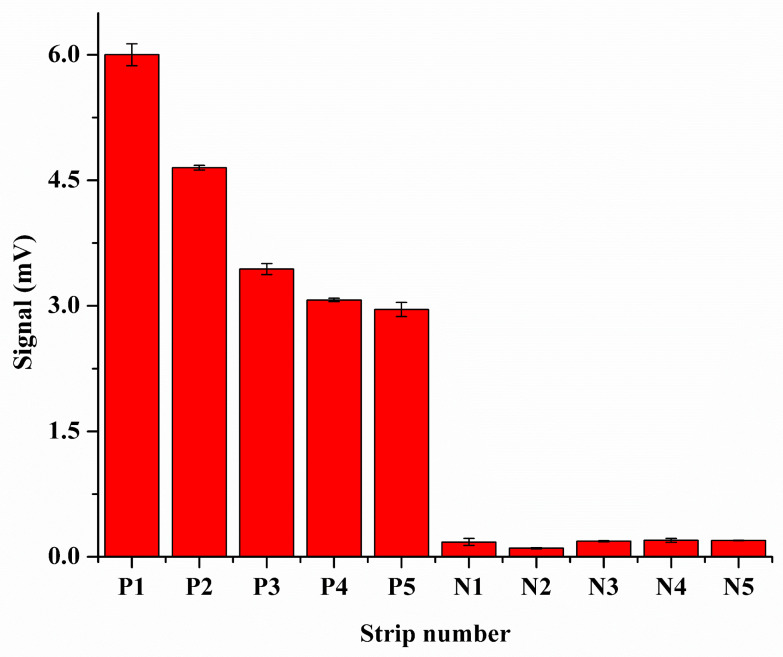
Fluorescence intensity of positive rat serum samples (rat serum samples with anti-SARS-CoV-2 spike IgG antibody, P1–P5) and negative rat serum samples (rat serum samples without anti-SARS-CoV-2 spike IgG antibody, N1–N5).

The usability of the quantum dot-doped LFIA strip was evaluated for simulated samples detection. Five kinds of positive serum specimens (P1–P5) and five kinds of negative serum samples (N1–N5) were detected. As illustrated in Figure 4, the fluorescence signal intensity of simulated positive serum specimens was much higher than negative samples. The detection rate of the simulated positive serum specimens was 100%, which revealed that the LFIA strip possessed good specificity. Additionally, as shown in Figure S5A,B, the SiO_2_@Au@CDs NP-based LFIA strip and AuNP-based LFIA strip possess similar properties in the detection of IgG by the naked eye. Meanwhile, the linearity of the strips for IgG detection was studied, and the SiO_2_@Au@CDs NPs-doped LFIA strip had good linearity ranging from 7.4 × 10^−7^ to 7.4 × 10^−4^ mg/mL (illustrated in Figure S6), while the SiO_2_@CDs strip showed good linearity ranging from 7.4 × 10^−5^ to 3.7 × 10^−2^ mg/mL. The results confirmed that the SiO_2_@Au@CDs NP-based LFIA strip possesses a larger linear range, and the SiO_2_@Au@CDs NP-based LFIA strip has an LOD of 1.2 × 10^−7^ mg/mL (LOD = 3σ/k). As exhibited in Table 1, the recoveries of the SiO_2_@Au@CD-based LFIA strips were better than those of the SiO_2_@CDs strips. Meanwhile, the results from this investigation were compared with those of the previously proposed approaches (Table 2) [4,44,45,46]. All the results demonstrated that the SiO_2_@Au@CD-based LFIA strips were suitable for anti-SARS-CoV-2 IgG quantitative determination. The probable reasons were as follows: first, the specific surface area of the SiO_2_ NPs was enhanced by the coating of Au NPs, and the capacity of CDs can be dramatically increased, which finally resulted in the fluorescence enhancement. Second, the fluorescence signal of the materials can be significantly enhanced via fluorescence resonance energy transfer between CDs and Au NPs [47,48]. Third, fluorescence enhancement efficiency can be greatly influenced by the size of the materials (the fluorescence emission can be enhanced by the metal particles) [49].

**Figure 5:**
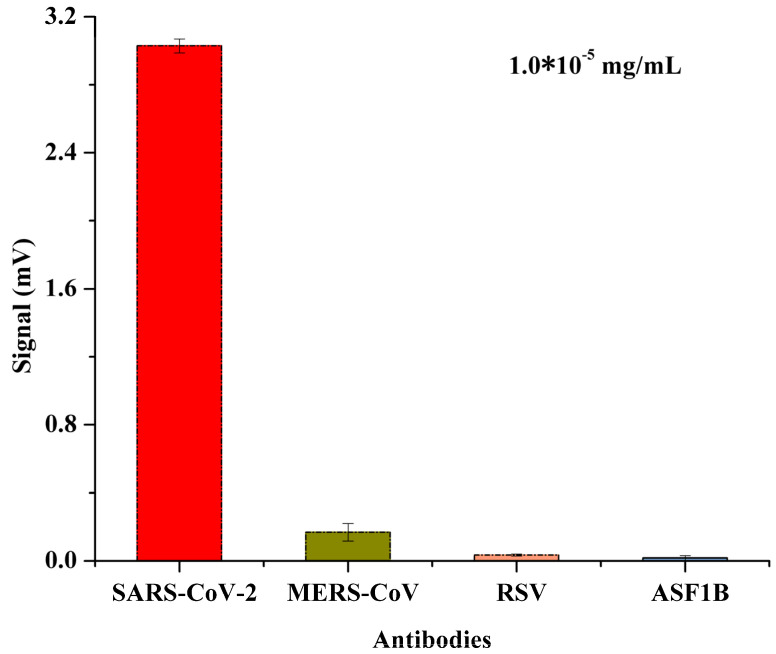
Specificity of SiO_2_@Au@CD-based LFIA strips.

Furthermore, the specificity, repeatability, accuracy, and stability of the LFIA were important parameters and were studied in depth. As depicted in Figure 5, serum samples containing several antibodies for respiratory viruses including anti-SARS-CoV-2 IgG (10 ng/mL), MERS-CoV IgG (10 ng/mL), RSV IgG (10 ng/mL), and ASF1B IgG (10 ng/mL) were used as targets to evaluate specificity of the LFIA. The fluorescence intensity of anti-SARS-CoV-2 IgG was much higher than other kinds of antibodies, suggesting that LFIA strips possess good specificity. As shown in Figure S5, the T line of the SiO_2_@Au@CDs LFIA strip for anti-SARS-CoV-2 IgG was clearer than SiO_2_@Au@CDs LFIA strips for other antibodies (MERS-CoV, RSV, ASF1B) and the blank sample without antibodies. This further indicated that SiO_2_@Au@CDs LFIA strips could be used by the naked eye and possessed good specificity.

**Figure 6:**
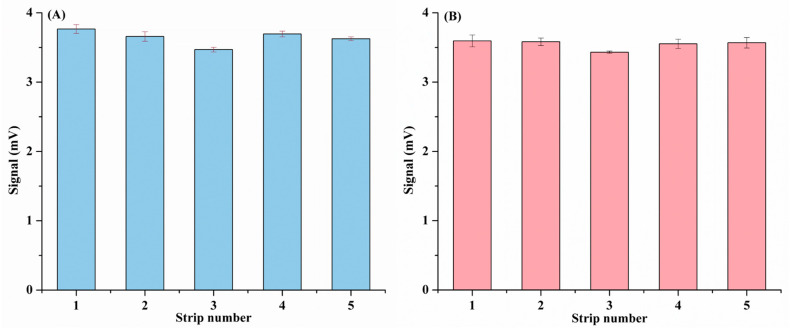
Intra-batch (**A**) and inter-batch (**B**) repeatability of SiO_2_@Au@CDs LFIA strip test (fluorescence intensity corresponding to T-line of 7.4 × 10^−5^ mg/mL anti-SARS-CoV-2 S1 antibody).

**Figure 7:**
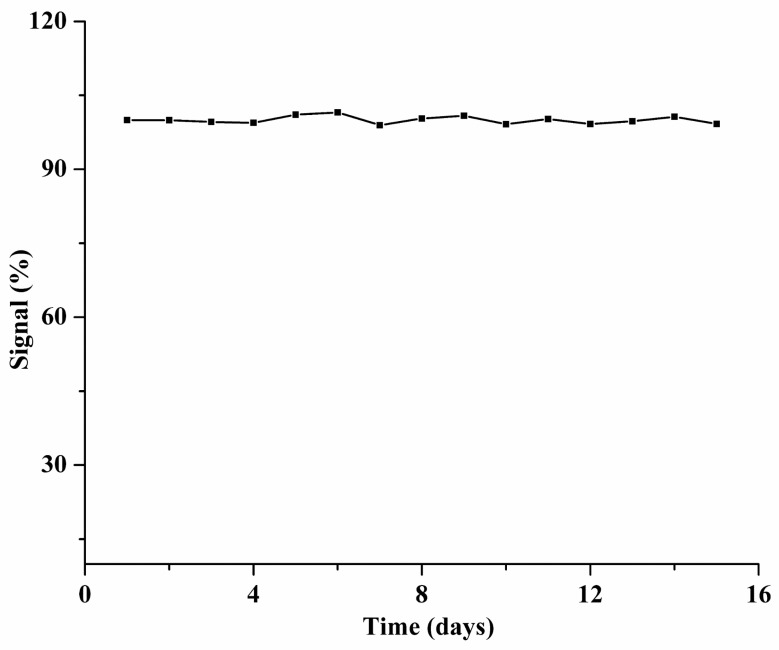
Stability of the SiO_2_@Au@CDs NPs stored in ethanol.

Additionally, as shown in Figure 6, the repeatability of the LFIA strip was fully validated by dropping samples with anti-SARS-CoV-2 IgG (7.4 × 10^−5^ mg/mL). The relative standard deviation of five different batches of LFIA strips and the same batch of LFIA strips tested five times was 3.02% and 2.30%, separately, which indicated that the resulting LFIA strips possessed high repeatability and accuracy. Moreover, as illustrated in Figure 7, the stability of the LFIA strip was investigated by immersing the SiO_2_@Au@CDs NPs in EtOH for 15 days. In the process, the fluorescence intensity of the SiO_2_@Au@CDs NP-based LFIA strip ranged from 101.55% to 99.17%. However, the stability of SiO_2_@Au@CDs in ethanol showed a random nature, which was probably caused by the researchers’ operation, and the specific steps have been added (Video S1 and Figure S4). Even so, the above results suggested that the materials possessed good stability.

## 4. Conclusions

Altogether, the fabrication of a novel SiO_2_@Au@CDs NP-based LFIA strip was first reported, and evaluated for the detection of SARS-CoV-2 IgG antibody. For the detection of SARS-CoV-2 IgG antibody, the SiO_2_@Au@CDs NP-based LFIA strip had the advantages of high sensitivity (1.2 × 10^−7^ mg/mL), speediness (15 min), wide 7.4 × 10^−7^~7.4 × 10^−4^ mg/mL (7.4 × 10^−7^~7.4 × 10^−4^ mg/mL), high accuracy, practical feasibility, and low cost. Moreover, the SiO_2_@Au@CDs NP-based LFIA strip had high specificity towards SARS-CoV-2 IgG antibodies. In order to improve sensitivity and reduce errors, it is necessary to develop automated reader devices. Additionally, LFIAs can help realize on-site detection of SARS-CoV-2, and patients can diagnose *COVID-19* via LFIAs at home. What’s more, the SiO_2_@Au@CDs NPs might be labeled with other proteins and applied for quantitative analysis of corresponding antibodies. Scientists who work in medicine, pharmacy, environmental science, and analytical chemistry may be interested in this work.

## Figures and Tables

**Table 1:** The properties of SiO_2_@CD-based LFIA strips and SiO_2_@Au@CD-based LFIA strips for anti-SARS-CoV-2 IgG detection.

Sample	Antibody (×10^−4^ mg/mL)	SiO_2_@CD-Based LFIA Strips		SiO_2_@Au@CD-Based LFIA Strips	
		(×10^−4^ mg/mL)	RC (%)	(×10^−4^ mg/mL)	RC (%)
1	7.4	5.95	80.4	7.37	99.6
2	3.7	4.03	108.9	3.91	105.7
3	1.85	1.51	81.6	1.64	89.1
4	0.74	0.69	93.2	0.68	91.9
5	0.37	0.34	91.9	0.30	81.1

**Table 2:** Several NP-based LFIA strips for fluorescence detection.

Nanoparticles	Target	Linear Range	LOD	Detection Time (min)	Reference
SiO_2_@Au/QD	S1 protein	0.05–1000 ng/mL	0.033 ng/mL	30	[4]
red emission-enhanced carbon dot (CD)-based silica spheres	SARS-CoV-2 nucleocapsid proteins	10 pg/mL–1 μg/mL	10 pg/mL	7	[44]
Ag@Au NPs-NCP	2019 Novel Coronavirus antibodies	0.1 pg/mL– 10 ng/mL	50 fg/mL	150	[45]
Si-Mn:ZnSe NPs	SARS-CoV-2 S protein	0.05–10 ng/mL and 5–50 ng/mL	0.032 ng/mL	60	[46]
SiO_2_@Au@CDs	IgG	7.4 × 10^−4^–0.74 ng/mL	1.2 × 10^−4^ ng/mL	15	This work

## Data Availability

The data will be made available on request.

## Supplementary Materials

The following supporting information can be downloaded at: https://www.mdpi.com/article/10.3390/biom14121568/s1, Figure S1: EDS spectra of the materials; Figure S2: UV-Vis absorption spectra of the materials; Figure S3: Fluorescence spectra of SiO_2_, SiO_2_@Au, SiO_2_@CDs, and SiO_2_@Au@CDs under UV light; Figure S4: Pictures of the fluorescence detection system; Figure S5: Images of (A) AuNPs-based LFIA strips, and (B) SiO_2_@Au@CDs LFIA strips for different concentrations of anti-SARS-CoV-2 S1 antibodies, (C) SiO_2_@Au@CDs LFIA strips for diverse antibodies; Figure S6: (A) the linearity of SiO_2_@Au@CDs (7.4 × 10^−7^~7.4 × 10^−4^ mg/mL) and (B) SiO_2_@CDs (7.4 × 10^−5^~3.7 × 10^−2^ mg/mL) based LFIA strips for IgG detection; Table S1: Quantification results of the materials via element EDS spectra; Table S2: Element analysis results of the materials; Video S1: The relevant video of the measurement process.
