# Supplementary material for: Fast and Sensitive Detection of Anti-SARS-CoV-2 IgG Using SiO2@Au@CDs Nanoparticle-Based Lateral Flow Immunoassay Strip Coupled with Miniaturized Fluorimeter
Source: Biomolecules. 2024 Dec 9;14(12):1568. doi: 10.3390/biom14121568 (PMC11673715; doi:10.3390/biom14121568)
Supplement: Supplementary file 1 [file biomolecules-14-01568-s001.zip › biomolecules-3277503-supplementary.pdf]

# Supporting Information

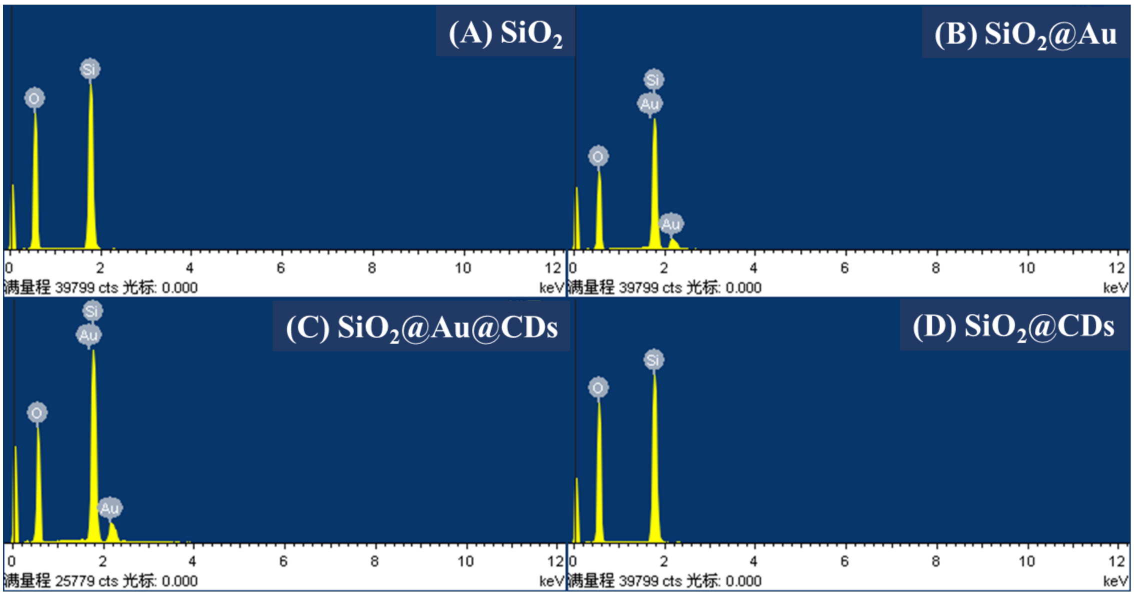


**Figure. S1** EDS spectra of the materials.

**Table S1** Quantification results of the materials via element EDS spectra.

| | O (weight, %) | Si (weight, %) | Au (weight, %) | totals |
|---|---|---|---|---|
| $SiO_2$ | 63.69 | 36.31 | | 100 |
| $SiO_2$@Au | 52.66 | 33.45 | 13.90 | 100 |
| $SiO_2$@Au@CDs | 51.05 | 32.22 | 16.72 | 100 |
| $SiO_2$@CDs | 63.95 | 36.05 | 0 | 100 |

**Table S2** Element analysis results of the materials.

| | N (weight, %) | C (weight, %) |
|---|---|---|
| $SiO_2$ | 0 | 0 |
| $SiO_2$@Au | 0 | 0.226 |
| $SiO_2$@Au@CDs | 0.025 | 0.195 |
| $SiO_2$@CDs | 0.053 | 1.067 |

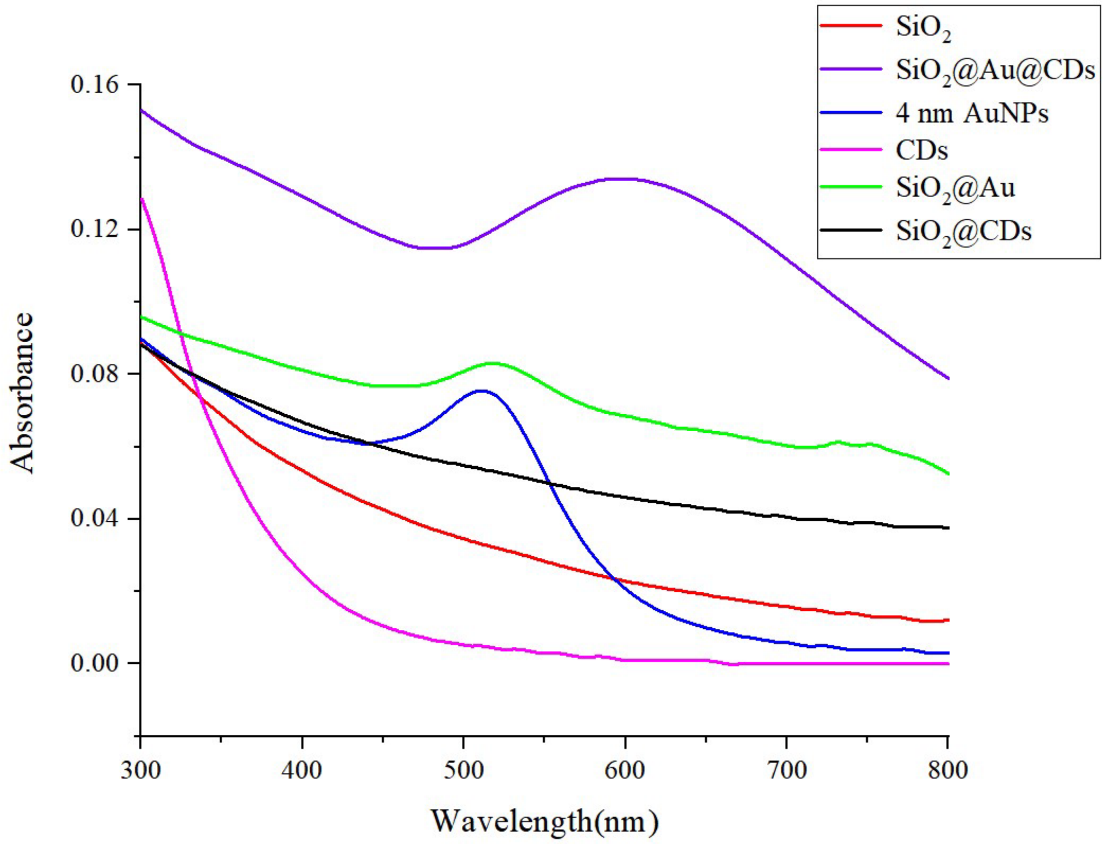


**Figure. S2** UV-Vis absorption spectra of the materials.

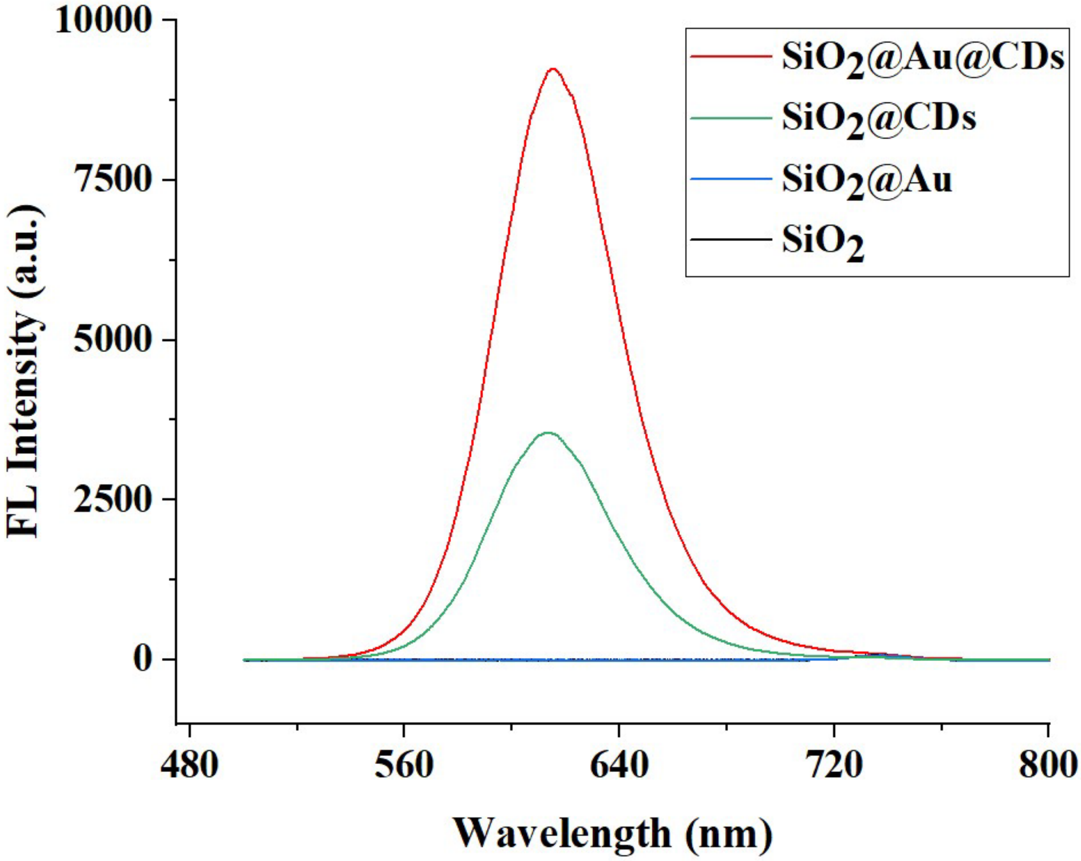


**Figure S3** Fluorescence spectra of $SiO_2$, $SiO_2$@Au, $SiO_2$@CDs, and $SiO_2$@Au@CDs under UV light.

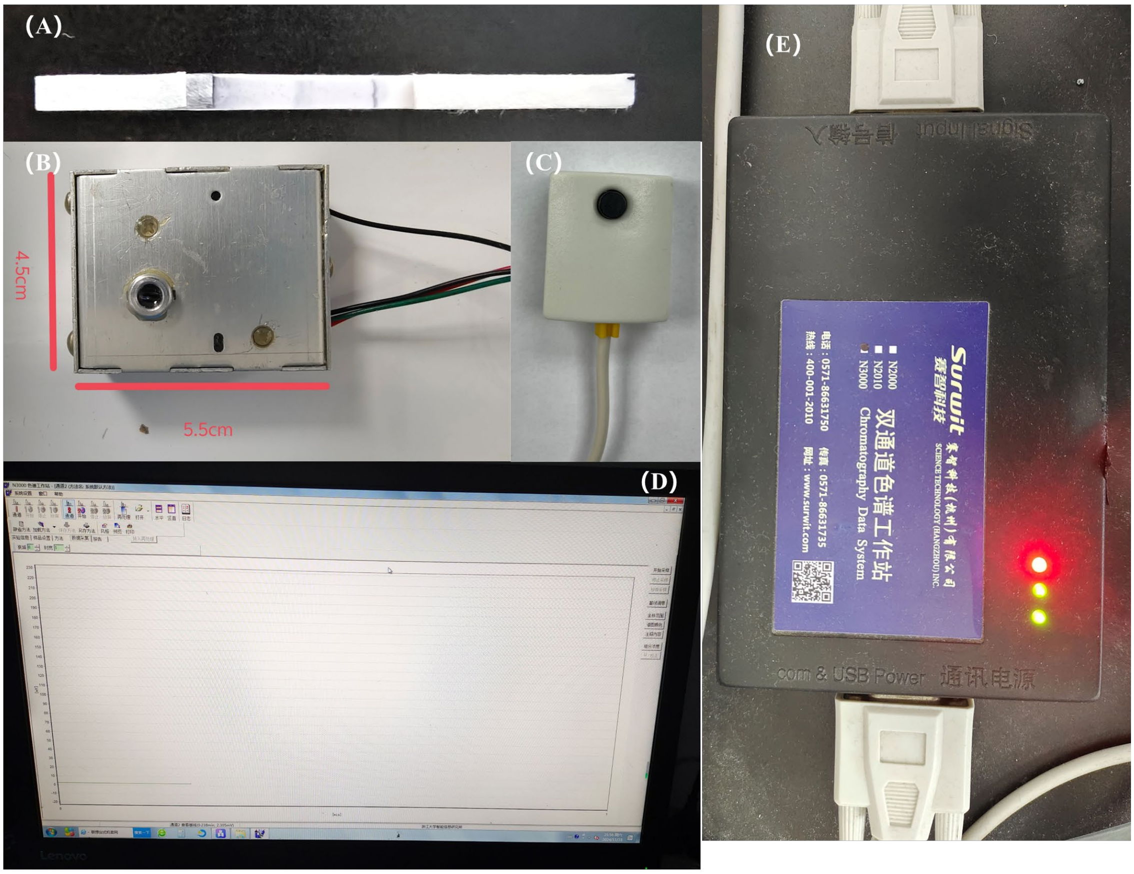


**Figure S4** Pictures of the fluorescence detection system. (A) LFIA strip, (B) miniaturized fluorimeter, (C) control button, (D) computer, (D) data collection box.

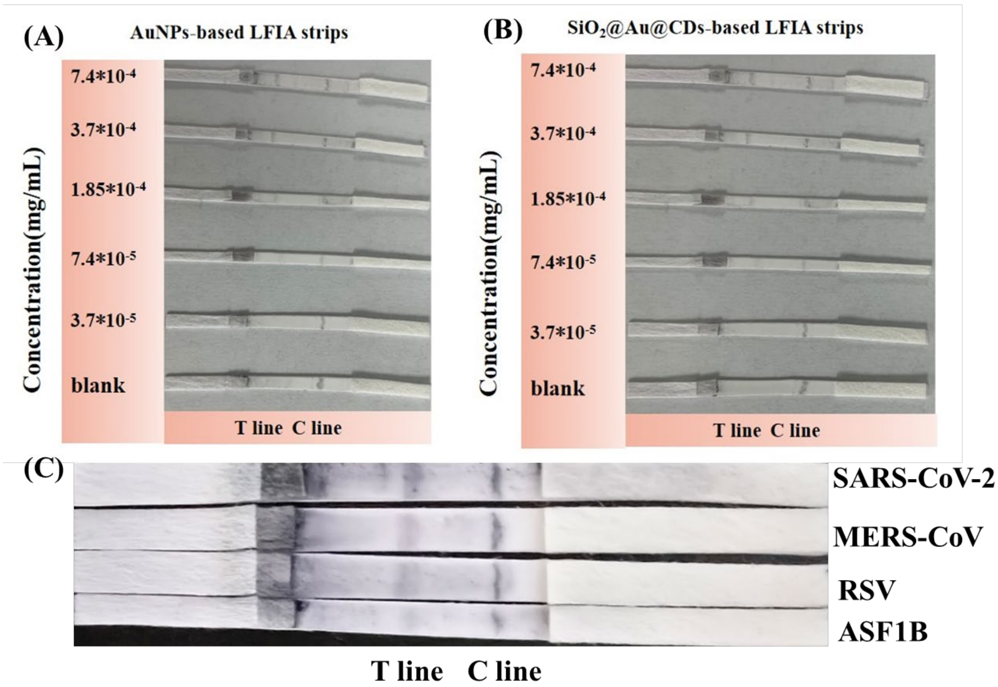


**Figure S5** Images of (A) AuNPs-based LFIA strips, and (B) $SiO_2$@Au@CDs LFIA strips for different concentrations of anti-SARS-CoV-2 S1 antibodies, (C) $SiO_2$@Au@CDs LFIA strips for diverse antibodies.

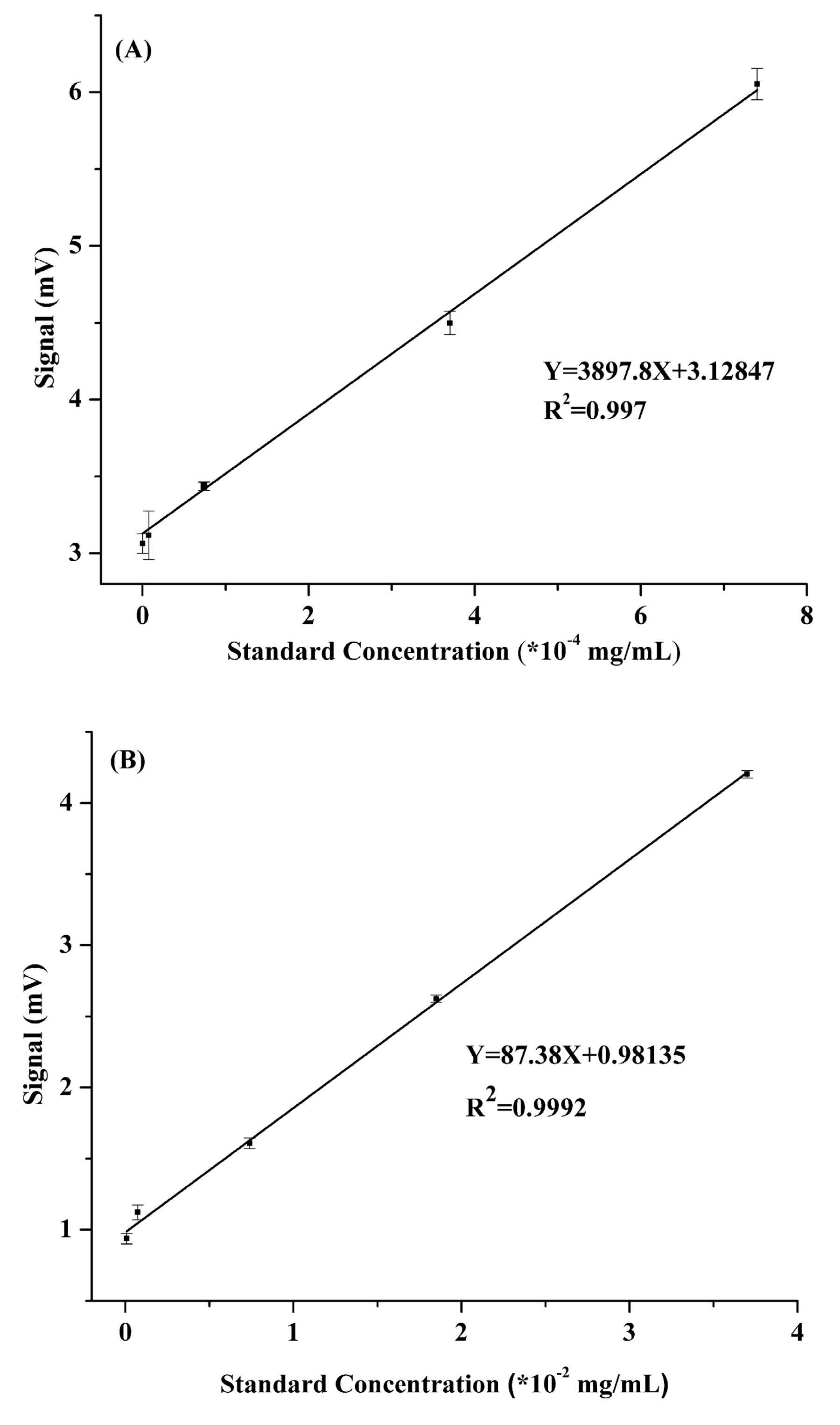


**Figure S6** (A) the linearity of $SiO_2$@Au@CDs ($7.4\times10^{-7}$~$7.4\times10^{-4}$ mg/mL) and (B) $SiO_2$@CDs ($7.4\times10^{-5}$~$3.7\times10^{-2}$ mg/mL) based LFIA strips for IgG detection.
